# Pivotal factors associated with the immunosuppressive tumor microenvironment and melanoma metastasis

**DOI:** 10.1002/cam4.3963

**Published:** 2021-06-22

**Authors:** Chuan Zhang, Dan Dang, Lele Cong, Hongyan Sun, Xianling Cong

**Affiliations:** ^1^ Department of Dermatology China‐Japan Union Hospital of Jilin University Changchun People’s Republic of China; ^2^ Department of Pediatric Surgery First Hospital of Jilin University Changchun People’s Republic of China; ^3^ Department of Neonatology First Hospital of Jilin University Changchun People’s Republic of China; ^4^ Department of Biobank China‐Japan Union Hospital of Jilin University Changchun People’s Republic of China

**Keywords:** IL2RA, IL7R, INFG, lncRNA, melanoma, miRNA, regulatory T cell, TNFRSF

## Abstract

**Background:**

Considering melanoma is the deadliest malignancy among dermatoma and presently lacks effective therapies, there is an urgent need to investigate the potential mechanisms underlying melanoma metastasis and determine prospective therapeutic targets for precise treatment of melanoma.

**Method:**

Hub genes in melanoma metastasis were identified by analyzing RNA‐seq data (mRNA, miRNA, and lncRNA) obtained from TCGA database. Then the identified hub genes were validated in human tissues with qRT‐PCR, followed by survival analysis. Competing endogenous RNAs of the hub genes were defined to clarify potential molecular mechanism of melanoma progression. Then central gene‐related signaling pathways were analyzed, followed by immune cell abundance analysis in tumor microenvironment with CYTERSORTx.

**Result:**

A tetrad of IL2RA, IL2RG, IFNG, and IL7R genes were determined as hub genes and verified by qRT‐PCR, which were significantly associated with unfavorable prognosis in melanoma. LINC02446, LINC01857, and LINC02384 may act as competing endogenous lncRNAs of IL2RA and IL7R through absorbing their shared miR.891a.5p and miR.203b.3p. JAK—STAT signaling pathway identified as the most relevant pathway in melanoma metastasis, as well as a wealthy of genes including TNFRSF 13B, TNFRSF17, TNFRSF9, TNFRSF8, TNFRSF13C, TNFRSF11B, LAG3, NRP1, ENTPD1, NT5E, CCL21, and CCR7, may induce tumor autoimmune suppression through enhancing regulatory T‐cell abundance and performance in the tumor microenvironment. And regulatory T‐cell proportion was indeed critically elevated in metastatic melanoma relative to primary melanoma, as well as in highly expressed IL2RA, IL2RG, IL7R, and IFNG group than their respective counterparts.

**Conclusion:**

Elevated IL2RA, IL2RG, IL7R, and IFNG expression may play a central role in promoting melanoma metastasis through up regulation of intratumoral regulatory T—cell proportion mainly by activation of JAK—STAT signaling pathway. LINC02446, LINC01857, and LINC02384 may stimulate melanoma progression by reducing tumor‐protecting miR.891a.5p and miR.203b.3p. A number of identified molecules including TNFRSF13B, LAG3, NRP1, ENTPD1, NT5E, CCL21, and CCR7 can serve as future therapeutic targets in melanoma treatment.

## Graphical abstract

Elevated IL2RA, IL2RG, IL7R, and IFNG expression may play a central role in promoting melanoma metastasis via increase in intratumoral regulatory T cell proportion, mainly by activation of the JAK–STAT signaling pathway. LINC02446, LINC01857, and LINC02384 may stimulate melanoma progression by reducing tumor‐protecting miR.891a.5p and miR.203b.3p. A number of identified molecules, including TNFRSF13B, LAG3, NRP1, ENTPD1, NT5E, CCL21, and CCR7, can serve as future therapeutic targets in the treatment of melanoma.

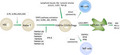

## 1 BACKGROUND

Cutaneous malignant melanoma as the most lethal form of skin cancer,^1^, ^2^ is characterized by a high metastasis rate, with a low survival rate of 27% in patients with metastatic melanoma.^3^ The incidence of melanoma is gradually increasing, especially in the Caucasian population, and melanoma is the leading malignancy and cause of cancer‐related death in Australians aged 15–44 years.^4^, ^5^ Immunotherapies, including CTLA‐4 and PD‐1/PD‐L1 inhibitors, are the first‐line therapeutic choice for metastatic melanoma.^6^, ^7^, ^8^ However, approximately half of melanoma patients treated with immunotherapies will develop primary or acquired resistance,^8^, ^9^, ^10^ which poses a big challenge for clinicians and patients. Therefore, it is urgent to investigate the underlying mechanism of melanoma metastasis and identify potential therapeutic targets for treatment of melanoma.

The Cancer Genome Atlas (TCGA)^11^ is currently the largest reservoir of cancer transcriptomes publicly accessible and freely available to medical researchers worldwide. TCGA database has molecularly profiled over 20000 primary cancer and matched normal samples spanning 33 cancer types. Over 2.6 petabytes of genomic, epigenomic, transcriptomic, and proteomic data are publicly available in TCGA database. The data has already contributed to advance in our competence to better understand cancer.^12^, ^13^, ^14^, ^15^

To interrogate the underlying metastatic mechanism of melanoma and identify potential therapeutic targets for melanoma, here we analyzed gene expressional profile of melanoma from the TCGA database, and validated the results with qRT‐PCR in human melanoma tissues. Furthermore, we utilized the CYBERSORTx,^16^ a digital cytometry that enables estimation of cell type abundances and cell‐type‐specific gene expression profiles from bulk tissue transcriptomes, to determine the crucial immune cell type and its gene expression profiles in advanced melanoma microenvironment. The present research will provide novel prospective therapeutic strategies for advanced melanoma, paving the way to future promising precision medicine in metastatic melanoma.

## 2 MATERIALS AND METHODS

### 2.1 Human samples

Three normal skin tissues and three melanoma tissues were derived from three patients with melanoma who were all operated in China‐Japan Union Hospital of Jilin University. All skin samples were stored at −80°C before total RNA was extracted.

### 2.2 Public datasets

A total of 472 melanoma samples with raw RNA‐seq reads (mRNA, miRNA, and lncRNA) and corresponding clinical information were garnered from TCGA database, including 105 primary melanoma patients and 367 metastatic patients. Single‐cell RNA‐seq data of immune cells were obtained from CIBERSORTx datasets.

### 2.3 RNA extraction and Quantitative Real‐time PCR

Total RNA was extracted from human tissues using TRIzol reagent (Invitrogen) following the manufacturer's protocols. cDNA was synthesized using the the GoScript Reverse Transcription System Kit (A5000; Promega). Real‐time PCR primers, including human β‐Actin (F: CATGTACGTTGCTATCCAGGC, R: CTCCTTAATGTCACGCACGAT), IFNG (F: CGTTTTGGGTTCTCTTGGCTG; R: TCTGTCACTCTCCTCTTTCCAAT), IL2RA (F: AGCTCTGCCACTCGGAACAC; R: TGCCCCACCACGAAATGAT), IL2RG (F: CTGGCTGGAACGGACGATG; R: TTGGGGGAATCTCACTGACG), and IL7R (F: CACAAAGCTGACACTCCTGC; R: GATCCATCTCCCCTGAGCTATT), were designed with Primer‐BlAST and checked by Oligo (Version 6.44). Quantitative Real‐time PCR analysis was performed using SYBR® Premix Ex Taq™ II (Tli RNaseH Plus) ROX plus (Takara) on a ABI 7500 Real‐Time PCR System (Applied Biosystems, CA, USA). Relative gene expression in each sample was calculated with the ‐ΔΔCt method.

### 2.4 Calculation of differentially expressed genes

All differentially expressed (DE) genes including mRNA, lncRNA, and microRNA are computed with raw data by the "edgeR" R package, which provides a procedure to normalize the raw data, filter the very low expression genes and calculate the DE genes between different groups.

### 2.5 Identification of key modules in all DE mRNAs

Functional protein—protein interaction (PPI) network was established based on functional enrichment analysis by STRING, an online tool.^17^ Next, PPI network result was further analyzed with MCODE, a plug‐in of Cytoscape software (version 3.6.0), to obtain the most relevant gene cluster. Key parameters are as follows: Include Loops: false, Degree Cutoff: 2, Node Score Cutoff: 0.2, Haircut: true, Fluff: false, K‐Core: 2, Max. Depth: 100.

### 2.6 KEGG pathway and Gene Ontology analysis

Gene set enrichment analysis (GSEA) for all genes expressed in melanoma were performed using GSEA software (version 3.0) and reexamined by DAVID Bioinformatics Resource 6.8.^18^

### 2.7 Identification of Hub genes in melanoma metastasis

The overlapping genes between the most relevant gene cluster identified by MCODE analysis and the most significant pathway in GSEA analysis results are determined as hub genes in melanoma metastasis.

### 2.8 Identification and construction of the ceRNA network

To further investigate the molecular mechanisms of hub genes, we established a competing endogenous RNA (ceRNA) network. The ceRNA theory is that any RNA transcript that contains MREs can sequester miRNAs from other targets sharing the same MREs, thereby regulating their expression.^19^, ^20^ Importantly, the ability of two transcripts to cross‐regulate each other can be bioinformatically predicted based on their individual expression levels.^19^ All DE miRNA, DE lncRNA and DE mRNA‐seq data were obtained from TCGA database, and normalized by voom method in the R package "limma." Pearson correlation coefficients between DE miRNA and DE mRNA, between DE miRNA and DE lncRNA, and between DE mRNA and DE lncRNA were computed, respectively. The correlation intensity was generally classified into five grades based on the absolute value of the correlation coefficient: 0.00–0.19 corresponds to very weak, 0.20–0.39 to weak, 0.40–0.59 to moderate, 0.60–0.79 to strong, and 0.80–1.0 to very strong.^21^ Hereon, miRNA‐mRNA pairs with r < −0.3 and *p* value < 0.05 are considered to have a significant association, and similarly miRNA‐lncRNA pairs with r < −0.3 and *p* value < 0.05, mRNA‐lncRNA pairs with |r|>0.6 and *p* value < 0.05 are considered to have a significant relationship.

Screen the above results for significantly paired lncRNA‐mRNA, both of which is significantly correlated to the same miRNA, to obtain a miRNA‐lncRNA‐mRNA network, that is ceRNA, among which lncRNAs can adsorb miRNAs via a "sponge effect," suggesting that miRNAs are competitively bound by both their corresponding lncRNAs and mRNAs.

### 2.9 Estimation of cell type abundance

Proportion of immune cell type in melanoma was calculated with CYBERSORTx, an online tool developed by a scientific team from Stanford University, which enables precise estimation of cell abundance with RNA‐seq data and corresponding single cell RNA‐seq data. The operating parameters used in the present study were as follows: B‐mode, disable quantile normalization, and permutation for significance analysis 100. We filtered out the samples with *p* > 0.05 to increase the accuracy of the estimated results.

### 2.10 Statistical analysis

Statistical analysis was mainly performed using R Studio (version 3.6.1). Continuous values were analyzed using *t*‐test. Survival analysis was performed using log‐rank test, and plotted using Kaplan—Meier survival curves. *p* < 0.05 was considered statistically significant.

## 3 RESULTS

### 3.1 1366 DE mRNAs between primary and metastatic melanoma were identified

**FIGURE 1:**
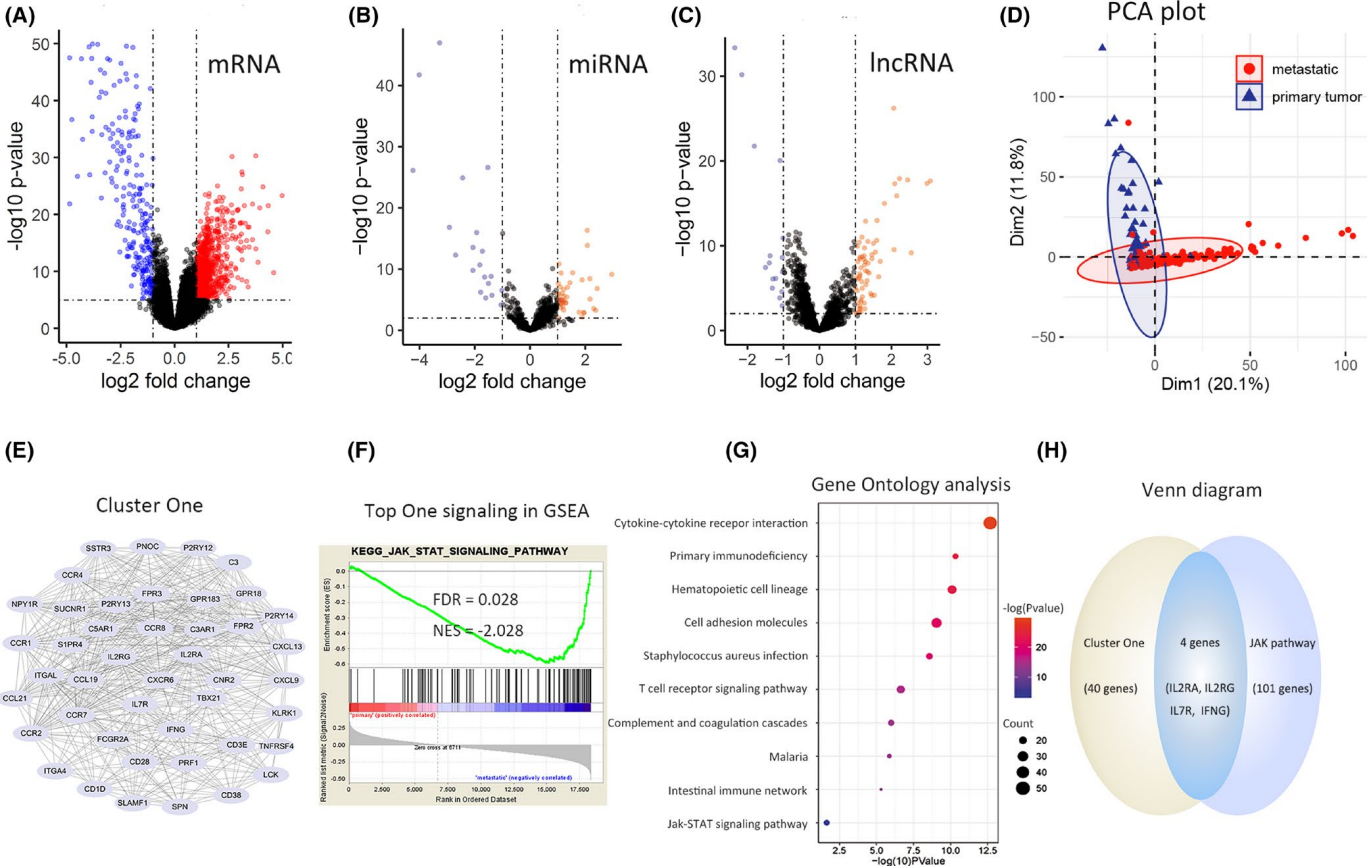
Brief schematic illustration of determination of hub genes. (A) 1366 DEmRNAs were associated with 55 396 genes expressed in melanoma, consisting of 998 up regulated genes (red dots) and 368 down regulated genes (blue dots) in advanced melanoma. (B) 68 DE miRNAs were obtained from 860 miRNAs in melanoma. (C) 81 DE lncRNAs were obtained from 2211 lncRNAs in melanoma. (D) The PCA plot displays a distinct gene expression mode between primary and metastatic melanoma. (E) The most relevant cluster from Cluster One in metastatic melanoma calculated by STRING and the MCODE plug‐in of Cytoscape, contains 45 DE genes that have a close functional correlation with each other. (F) Results of GSEA, based on RNA‐seq reads of 18 353 genes filtered from all 55 396 genes by eliminating extremely low‐expressed genes, determined that the JAK‐STAT signaling pathway was the top KEGG pathway, comprising 105 genes expressed in melanoma. (G) Results of Gene Ontology analysis of 998 upregulated genes showed that the JAK‐STAT pathway is one of the relevant signaling pathways in advanced melanoma. (H) Four overlapping genes were eventually selected as key genes via intersecting the genes in Cluster One and those in the JAK‐STAT pathway

To investigate the potential crucial genes involved in melanoma metastasis, we initially perform a DE RNA analysis (mRNA, miRNA, and lncRNA) on RNA‐seq data from TCGA via the R package "edgeR." There were divergent gene expression patterns between two groups, and a total of 1366 DE mRNAs, including 998 upregulated genes and 368 downmodulated genes, were identified (*p* < 0.05, |log_2_FC|>1) in metastatic melanoma compared with primary melanoma (Figure 1A‐D).

### 3.2 IL2RA, IL2R, IFNG, and IL7R genes were determined as hub genes in melanoma metastasis

To further detect the key genes in melanoma metastasis, we performed a series of bioinformatics analyses. Upregulated DE mRNAs were analyzed with online STRING tool to determine PPI network, and then PPI network was further categorized into 25 functionally specific clusters using MCODE plug‐in of Cytoscape. The most significant gene set, that is, Cluster One, consisted of 45 DE mRNAs (Figure 1E).

To interrogate the most relevant signaling pathway associated with melanoma, we performed GSEA for RNA‐seq data of all genes in melanoma, and performed the KEGG analysis for upregulated DE mRNAs using DAVID. The JAK—STAT signaling pathway containing 105 genes was identified as the most significant pathway ranking the first in GSEA results, and also present in DAVID results (Figure 1F‐G).

To obtain hub genes correlated with advanced melanoma, we intersected the genes present both in Cluster One and in JAK—STAT pathways, and four overlapping genes were obtained (Figure 1H).

### 3.3 **IL2RA, IL2R, IFNG, and IL7R were also significantly upregulated in melanoma tissues relative to normal tissues, and had a critically positive correlation to unfavorable prognosis in melanoma**.

**FIGURE 2:**
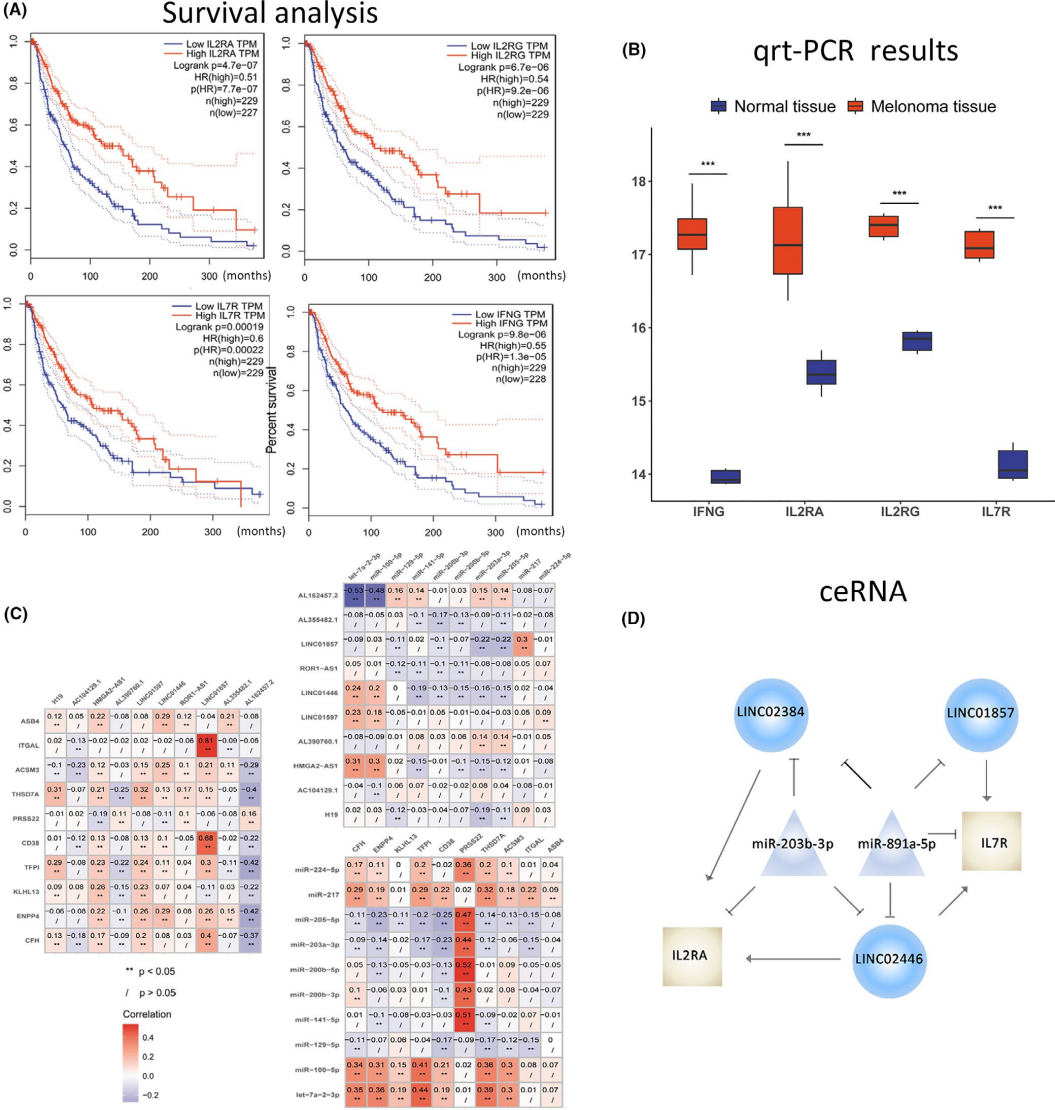
Further demonstration of selected pivotal genes supports their crucial role in the progression and prognosis of melanoma. A ceRNA (miRNA‐lncRNA‐mRNA) network involved in melanoma was identified. (A) The results of survival analysis display a remarkable distinction in survival between the *IL2RA* ^low^ and *IL2RA* ^high^ groups, between the *IL2RG* ^low^ and *IL2RG* ^high^ groups, between the *IL7R* ^low^ and *IL7R* ^high^ groups, and between the *IFNG* ^low^ and *IFNG* ^high^ groups. (B) RT‐PCR results of the four genes in human tissues show evident bias in mRNA expression between normal tissues and matched melanoma tissues. *represents a significant statistical difference. (C) Parts of the correlation analysis between DE miRNA and DE mRNA, between DE miRNA and DE lncRNA, and between DE mRNA and DE lncRNA are presented. (D) *IL7R*‐associated and *IL2RA*‐associated ceRNAs are displayed in the picture. T shape represents an inhibitory effect, while arrow shape represents an activating effect

To investigate whether the distinction also exists between normal and melanoma tissue, we tested the tetrad of IL2RA, IL2R, IFNG, and IL7R transcriptional expression between normal samples and melanoma samples with RT‐PCR, and observe the same expression pattern as expected (Figure 2B).

Furthermore, survival analysis for the four genes in melanoma were performed, and the results showed they were all significantly correlated to poor prognosis (Figure 2A).

### 3.4 lncRNA‐miRNA‐mRNA network

To explore the underlying molecular mechanisms of the four pivotal genes affecting melanoma progression, we next identified the four gene‐associated miRNA and lncRNA with Pearson correlation analysis in (Figure 2C). A total of 565 groups of ceRNAs were obtained from 68 DE miRNAs, 81 DE lncRNA, and 1285 DE mRNAs (Table S1). hsa.miR.203b.3p was negatively associated with LINC02446, LINC01857, LINC02384, and IL2RA, respectively, while LINC02446, LINC01857, and LINC02384 were all positively related to IL2RA, indicating that hsa.miR.203b.3p may suppress IL2RA expression and subsequently prohibit tumor progression, while LINC02446, LINC01857, and LINC02384 may promote IL2RA levels and thus stimulate cancer metastasis. hsa.miR.891a.5p was negatively associated with LINC02446, LINC01857, and IL7R, individually, while LINC02446 and LINC01857 were positively related to IL7R, implying that hsa.miR.891a.5p may inhibit IL7R expression and subsequently restrain tumor proliferation, whereas LINC02446 and LINC01857 may enhance IL7R abundance and thus boost cancer progression (Figure 2D). Notably, LINC01857 and LINC02446 were the shared competing lncRNA of both IL2RA and IL7R, supportive of their significant involvement in tumor progression. No significant ceRNA about IL2RG and IFNG were identified hereon.

### 3.5 IL2RA, IL2R, IFNG, and IL7R induce tumoral immune tolerance through stimulating function and proportion of regulatory T cells

**FIGURE 3:**
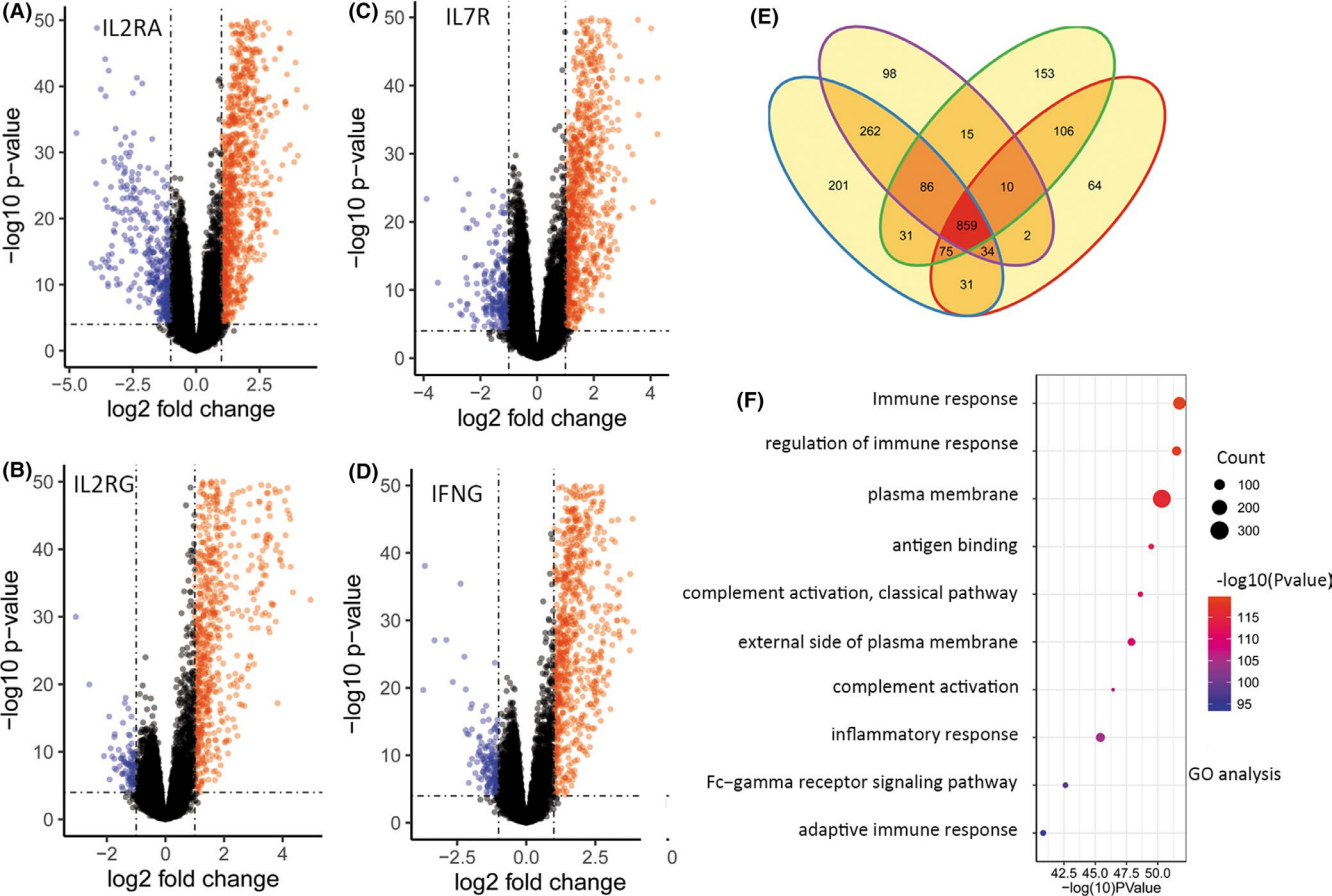
A number of critical genes, as well as regulatory T cells, may play a crucial role in tumor immune escape. (A‐D) We identified 1335 DE mRNAs between the *IL2RG* ^high^ and *IL2RG* ^low^ groups in melanoma, 1579 DE mRNAs between the *IL2RA* ^high^ and *IL2RA* ^low^ groups, 1366 DE mRNAs between the *IL7R* ^high^ and *IL7R* ^low^ groups, and 1181 DE mRNAs between the *IFNG* ^high^ and *IFNG* ^low^ groups. (E) A total of 859 overlapping genes were identified among DE mRNAs of the *IL2RA*, *IL2RG*, *IL7R*, and *IFNG* groups, accounting for a majority of DE mRNAs, indicating that the four pivotal genes have highly similar expression patterns and highly functional correlations. (F) Gene Ontology analysis of 859 overlapping genes revealed that the top 10 pathways were almost all related to immune response

**FIGURE 4:**
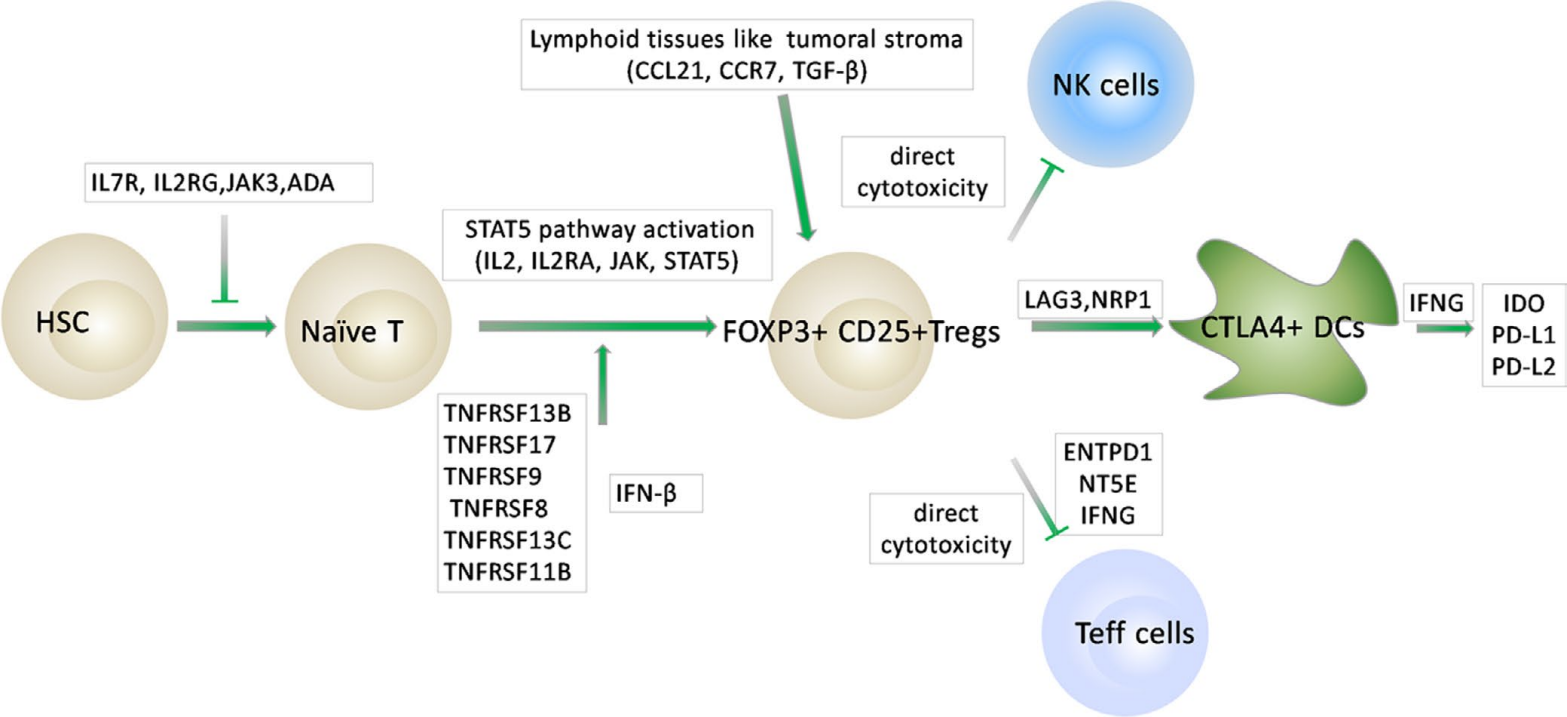
By investigating the differentially expressed genes involved in immune pathways in the literature, we extracted a number of highly relevant genes in melanoma metastasis, some of which were confirmed in previous studies, some of which are contradictory and not yet fully understood, and some of which were previously unpublished. T shape represents an inhibitory effect, while arrow shape represents an activating effect

To further interrogate hub gene‐related biological process and pathways in melanoma, we calculated the DE mRNAs between IL2RA^high^ group and IL2RA^low^ group, that between IL2RG^high^ group and IL2RG^low^ group, that between IL7R^high^ group and IL7R^low^ group, as well as that between IFNG^high^ group and IFNG^low^ group, respectively (Figure 3A‐D). Then the four results were intersected to obtain a subset of 859 genes (Figure 3E), which were subsequently analyzed using DAVID (Figure 3F). We observed that top 10 pathways were almost all immune related. To further identify the key genes, we investigated the involved genes in these immune‐associated pathways with literature and KEGG website. As a result, LAG3, NRP1, NT5E, CCL21, CCR7, and tumor necrosis factor receptor super family (TNFRSF) including TNFRSF 13B, TNFRSF17, TNFRSF9, TNFRSF8, TNFRSF13C, TNFRSF11B, and so on, were found significantly overexpressed in metastatic melanoma, and may involve autoimmune suppression in the tumor microenvironment and induce tumor evasion. Meanwhile, regulatory T (T_reg_) cells were considered as a pivotal immune cell type in advanced melanoma (Figure 4).

### 3.6 **Critically elevated levels of T_reg_ cells existed in metastatic melanoma compared with primary melanoma**.

**FIGURE 5:**
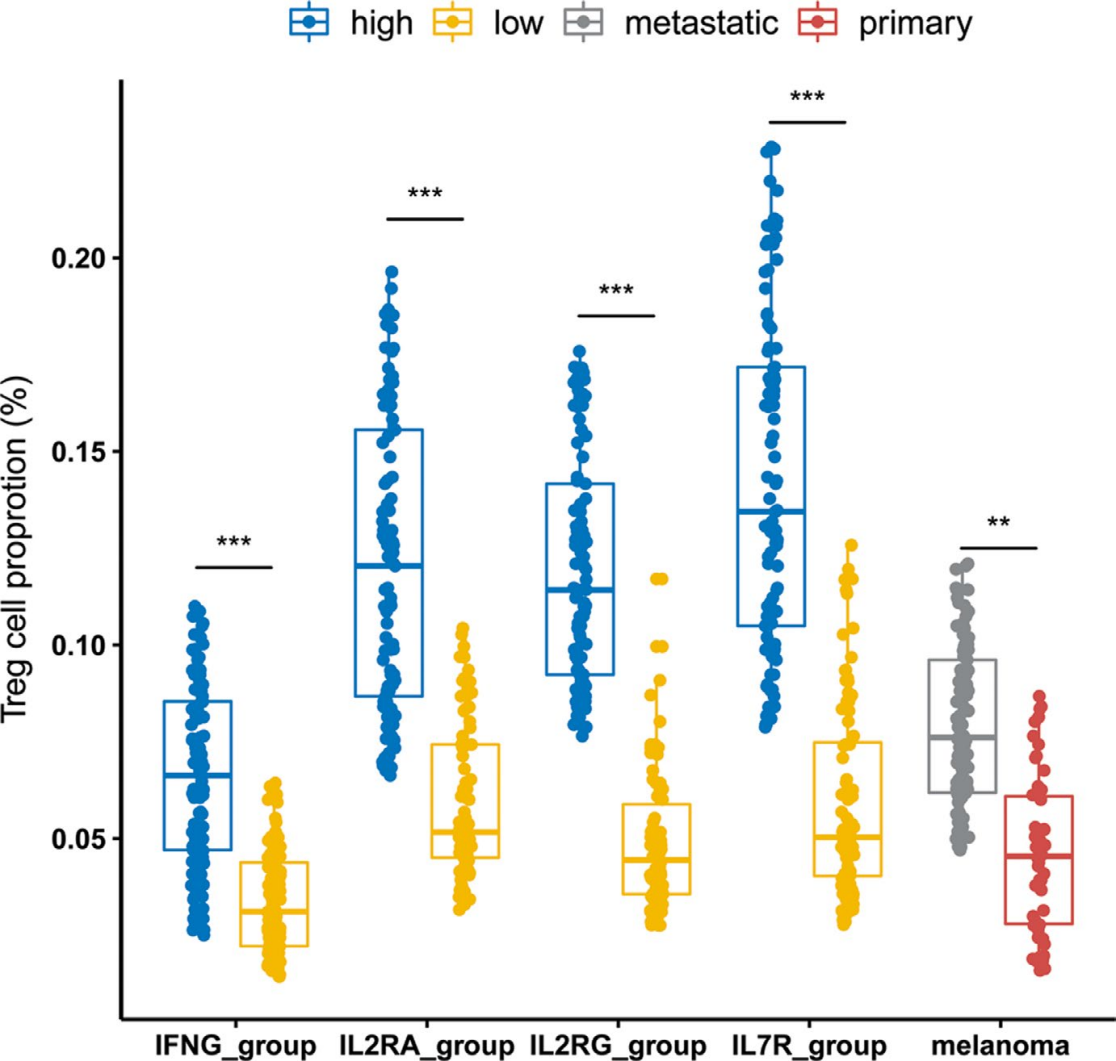
Significantly higher abundance of regulatory T cells was observed in advanced melanoma relative to primary melanoma as well as in the *IL2RA* ^high^ subgroup compared with the *IL2RA* ^low^ subgroup, in the *IL2RG* ^high^ subgroup compared with the *IL2RG* ^low^ subgroup, in the *IL7R* ^high^ subgroup compared with the *IL7R* ^low^ subgroup, and in the *IFNG* ^high^ subgroup compared with the *IFNG* ^low^ subgroup

To test whether T_reg_ cells are distinctively expressed in metastatic melanoma, we estimated the immune cell abundance in the tumor microenvironment by CYTERSORTx, and observed a significant elevation of T_reg_ cell proportion in advanced melanoma compared with primary melanoma, in IL2RA^high^ relative to IL2RA^low^ subgroup, in IL2RG^high^ subgroup compared with IL2RG^low^ subgroup, in IL7R^high^ subgroup compared with IL7R^low^ subgroup, and in IFNG^high^ subgroup compared with IFNG^low^ subgroup, respectively (Figure 5).

To explore the immune cell‐specific genes involved in advanced melanoma, we calculated DE genes in each immune cell type between primary and metastatic melanoma. The findings showed that ZNF775, GBA, ALKBH5, ZFYVE1, and MIEF2 were significantly upregulated in T_reg_ cells of metastatic melanoma compared with primary melanoma (Table 1).

**TABLE 1:** DE genes of each cell type between primary and metastatic melanoma

	Primary melanoma	Metastatic melanoma
CD8 T cells	–	C1QC, HLA.A, C1QB, CTSS, VSIG4
CD4 activated T cells	LSM8, MKLN1, RND1, HDAC8, WDPCP	C15orf48, PSMB10, TIMD4, IL15RA, RAC2
CD4 memory T cells	INPP4B, NATD1, ZNF517, STK3, CDC25B	ZNF26, PHKB, RBM26.AS1, SATB1, MBD5
T_reg_ cells	–	ZNF775, GBA, ALKBH5, ZFYVE1, MIEF2
T helper cells	–	HSPE1, NOP2, DERA, SFXN4, SLC25A19
M1 cells	AC083899.1, LRRC4C, VENTX, ANKRD31, CSRNP3	ANKIB1, FMR1, SMIM7, CAB39, ZFR
M2 cells	PSMC2, DDIT3, CTU2, ARMC10, PLOD3	CD300LG, TMEM80, C12orf76, ZFP2, DUSP7
NK cells	GRM2, PACSIN1, LINC01215	PFN4, HPCA, RPSAP9,
B cells	DNM30S, QTRT1, TRMT1, RPSAP54, PMFBP1	PRMT9, AMN1, PRAGB, TIPARP‐AS1

The table shows part of upregulated DE genes of specific immune cells in melanoma

## 4 DISCUSSION

We demonstrated that the upregulated critical molecules, pathways and cells in metastatic melanoma were all immune‐related, supportive of the dominant role of immune function in the process of melanoma metastasis. Overexpressed IL2RA, IL2RG, IFNG, and IL7R were identified as the hub genes associated with melanoma metastasis, and may promote melanoma progression through activation of JAK—STAT signaling pathway. The related genes, including TNFRSF13B, TNFRSF17, TNFRSF8, CCR7, NRP1, ENTPD1, LAG3, NRP1, TOX, and FOXP3, were also significantly upregulated and remarkably correlated to undesired prognosis in metastatic melanoma. These factors contributed to the augment in the abundance and function of T_reg_ cells in the tumor microenvironment, exacerbating tumor immune escape. In addition, we revealed that LINC01857, LINC02446, and LINC02384, as competing endogenous RNAs (ceRNAs) of IL2RA and IL7R, were oncogenes, while miR‐203b‐3p and miR‐891a‐5p, as microRNA sponges of IL2RA and IL7R, respectively, act as potential tumor suppressor molecules. Collectively, these findings provide insights to the underlying mechanisms of melanoma metastasis and help to establish promising molecular targeted therapy.

Our study revealed that IL2RA、IL2RG, IFNG, and IL7R were hub genes in melanoma metastasis and involved in JAK—STAT signaling pathway that was identified as an central signaling pathway in metastatic melanoma, which was rarely illustrated in previous studies.^22^, ^23^, ^24^, ^25^ Comparable results are observed with Non‐Hodgkin's lymphoma, in which activation of IL2Rγ—JAK3—STAT5 signaling pathway significantly promotes cancer progression while knockdown of IL2Rγ garners the same effect as Tofacitinib, a JAK inhibitor that effectively inhibits the activity of JAK1 and JAK3.^26^, ^27^ Actually, Julianna Volkó et al. found the assembly of IL2R may occur and exert its function in the cytoplasm, which is a probable factor in immunotherapy resistance,^28^ as IL2Rβ and IL2Rγ could form ligand‐independent homodimerization and stimulate mutant JAK3‐STAT pathway.^29^ However, there still exist no reports on whether IL2RG is abnormally expressed and what influence ectopic IL2RG expression levels may exert in melanoma. As for IL7R, it is reported that activated IL7R in lymphocytes induces activation of a set of genes, all of which have been reported as transcriptional targets of the JAK—STAT pathway,^30^ further implying aberrant IL7R expansion could lead to activation of JAK—STAT signaling pathway and subsequent proliferation of T_reg_ cells. In addition, restricted proportion and function of naive T cells by abnormally elevated IL7R can subsequently lead to a reduced activator T cells of functional immune response,^31^ underlying another potential mechanism of tumor immune evasion. Furthermore, activation of IL7Rα and γc heterodimeric complex stimulates JAK3‐STAT5 signaling pathway, driving hematological cancer development,^32^ implicating therapeutic relevance of IL7R as a therapeutic target.^33^ IFNG was another key gene identified to correlate with poor outcomes in melanoma in our study. Although production of IFNγ by T and NK cells in cancer microenvironment was required for cytotoxic activity,^34^ IFNγ may serve as a pivotal molecule to facilitate tumor tissue homeostasis, promoting cancer evasion.^35^ Moreover, IFNγ stimulates immunosuppressive IDO, PD‐L1, and PD‐L2 production,^36^, ^37^, ^38^, ^39^ to attenuate the cytotoxic response. Consistent with previous studies, we observed critically elevated IFNG expression levels present in advanced melanoma were significant correlated with undesired outcomes and reduced immune response in tumor stroma. Collectively, our findings underscored the core position of IL‐2RA, IL‐2RG, IFNG, IL‐7R, and JAK—STAT pathway in melanoma metastasis.

We also found that LINC01857, LINC02446, and LINC02384, as protective factors of IL2R and IL7R, were potential oncogenic molecules, while miR‐203b‐3p and miR‐891a‐5p were suppressive of IL2RA and IL7R respectively. At present, there are still no reports about LINC01857 in malignant melanoma, and three reports indicating that LINC01857 was a potential oncogene in glioma,^40^ breast cancer ^41^ and pancreatic cancer.^42^ At the same time, there are no related literature on LINC02446 and LINC02384. And miR‐891a‐5p is also a newly discovered microRNA, with only one paper showing its tumor‐promoting activity,^43^ which contradicts our finding. Over‐expressed miR‐203b‐3p was associated with favorable therapy response and patient survival in breast tumors,^44^ which was consistent with our observation.

Another relevant finding in this study was that overexpressed T_reg_ cells in metastatic melanoma as compared with that in primary melanoma, were the pivotal intermediate components by which IL2RA, IL2RG, IL7R, and IFNG exert their immunosuppressive effect. Collective literature indicated a poor prognosis for cancers with an abundance of intratumoral FOXP3^+^ T_reg_ cells,^45^, ^46^ whereas a minority of studies showed an advantageous survival in colorectal tumors with FOXP3^+^ T‐cell infiltrates.^47^ Here we displayed an unwanted prognosis in patients with high levels of FOXP3^+^ T cells. Tumor‐resident T_reg_ cells were reported to promote tumor expansion and progression by impairing antitumor immune response, subsequently stimulating tumoral angiogenesis ^46^, ^48^ and promoting metastasis through activation of NF‐κB signaling pathway.^49^ In effect, T_reg_ subpopulations are heterogeneous, and different T_reg_ subpopulations may have opposing effects on tumor progression.^50^, ^51^, ^52^, ^53^ Moreover, T_reg_ cells in the melanoma tumor microenvironment are driven by CD8^+^ T cells,^54^ which indicates that patients with elevated T_reg_ cells could have augmented CD8^+^ T cells simultaneously, thus having an improved survival.

Our study has several limitations. For the main body of the observation were obtained through bioinformatics means, further biological assays and clinical researches are warranted to support the results. Meanwhile, further details on association of T_reg_ cells with immune escape of melanoma are required to establish future precision medicine. Briefly, the present study is only a minor step on the long way to conquer melanoma, and is by no means an end.

## 5 CONCLUSIONS

In summary, we demonstrated that elevated IL2RA,IL2RG, IL7R, and IFNG expression may facilitate melanoma metastasis through upregulation of tumor‐resident T_reg_‐cell proportion by activation of JAK—STAT signaling pathway, and determined four hub gene‐associated ceRNAs, as well as functionally metastasis‐relevant molecules including TNFRSF13B, TNFRSF17, CCR7, NRP1, NRP1, TOX, FOXP3, LAG3, NRP1, and so on. Our findings should highlight immunosuppressive mechanisms underlying melanoma metastasis and provide insights into resistance rationale of immunotherapy on melanoma, paving the way to prospective targeted therapy of advanced melanoma.

## CONFLICT OF INTERESTS

The authors report no conflict of interest in this work.

## AUTHORS CONTRIBUTION

Chuan Zhang, Xianling Cong are responsible for the literature review and writing the discussion and introduction of the paper. Dan Dang, Lele Cong, Hongyan Sun are responsible for the bioinformatics analysis, material and methods and results sections of the manuscript.

## ETHICS STATEMENT

The study was approved by the Ethics Committee of China‐Japan Union Hospital of Jilin University. Written informed consent was obtained from patients.

## Supporting information

Table S1

## Data Availability

The datasets analyzed during the present study are available in the TCGA database.
